# Optimization of Extraction Buffer Conditions for Improved Heparin Affinity-Based Quantification of Bovine Lactoferrin in Various Dairy Products

**DOI:** 10.1093/jaoacint/qsag001

**Published:** 2026-01-08

**Authors:** Tomoaki Fukushima, Keisuke Yamamoto, Yukino Sato, Tatsuya Nojima, Mirei Odaka, Norichika Nishida, Hiroshi Ochi

**Affiliations:** R&D Division, Morinaga Milk Industry Co., Ltd, 5-1-83, Zama, Kanagawa, 252-8583, Japan; R&D Division, Morinaga Milk Industry Co., Ltd, 5-1-83, Zama, Kanagawa, 252-8583, Japan; R&D Division, Morinaga Milk Industry Co., Ltd, 5-1-83, Zama, Kanagawa, 252-8583, Japan; R&D Division, Morinaga Milk Industry Co., Ltd, 5-1-83, Zama, Kanagawa, 252-8583, Japan; R&D Division, Morinaga Milk Industry Co., Ltd, 5-1-83, Zama, Kanagawa, 252-8583, Japan; R&D Division, Morinaga Milk Industry Co., Ltd, 5-1-83, Zama, Kanagawa, 252-8583, Japan; R&D Division, Morinaga Milk Industry Co., Ltd, 5-1-83, Zama, Kanagawa, 252-8583, Japan

## Abstract

**Background:**

Bovine lactoferrin (bLF) has been supplemented to a variety of foods, particularly dairy products, to promote human health. Reliable analytical methods are required for accurate quantification of bLF in foods. Isolation of bLF from foods using a heparin column and quantification via HPLC have been reported (heparin method); however, its applicability remains limited.

**Objective:**

To develop a broadly applicable method for determining bLF in dairy products by optimizing the extraction buffer conditions used in the heparin method.

**Method:**

bLF was spiked into different dairy products and extracted using buffers with or without additives, such as urea, sodium chloride, EDTA, and Tween 20. These additives reduced interactions between bLF and dairy components and improved the interaction of bLF to the heparin column, with the buffer pH adjusted to 6.0. The extracted bLF was isolated using a heparin column and quantified via C4 reversed-phase HPLC using an external standard calibration curve.

**Results:**

The optimized extraction buffer improved bLF recovery rates across all tested dairy product matrixes.

**Conclusion:**

An improved extraction buffer was developed that enhanced the applicability of bLF quantification using the heparin method. This optimized extraction buffer enabled reliable recovery of bLF from diverse and complex dairy product matrixes.

**Highlight:**

The optimized extraction buffer significantly improves the recovery and quantification of bLF in various dairy products, providing a versatile solution for accurate bLF analysis.

Lactoferrin (LF) is a mammalian glycoprotein with a molecular weight of about 80 kDa that is secreted in milk, saliva, tears, and nasal secretions. LF is composed of two homologous domains (lobes), and each domain harbors an iron-binding site (1). LF has been reported to exert multiple physiological and nutritional functions, including roles in iron metabolism, immunomodulation, and antioxidant activity (2). Currently, bovine LF (bLF) is industrially produced from cow’s milk on a commercial scale and is approved as an ingredient or food additive in many countries (3). Due to its beneficial properties, bLF has been supplemented into various foods, particularly dairy products, to promote human health. Therefore, a versatile and accurate method for determining bLF in these products is needed.

Various methods for the analysis and quantification of bLF have been reported, including ELISA (4, 5), capillary electrophoresis (6), HPLC (7), LC–MS (8), and surface plasmon resonance biosensor immunoassay (9–11), with the latter recently adopted as an official AOAC Method. Among the available methods, HPLC is advantageous due to its use of widely available equipment and high reproducibility. However, it is practically difficult to directly determine the amount of bLF in food matrixes because other components may exhibit unpredictable behavior during HPLC analysis, such as peak overlap, co-elution with bLF, or other forms of interference. Therefore, separation and isolation of bLF from these interfering components are necessary to ensure accurate quantification.

Heparin is a negatively charged polysaccharide capable of binding the positively charged basic protein bLF across a wide pH range, not only through electrostatic interactions but also through specific affinity interactions that depend on the amino acid sequence of bLF (12, 13). The use of a heparin column to isolate bLF from various food matrixes followed by quantification with reversed-phase HPLC—referred to as the heparin method—was originally reported by Zhang et al. (14). This technique has been adopted in the Chinese National Standards (15) and is under consideration as an international standard (16) for bLF analysis in dairy products, such as infant formula. In the reported heparin method, bLF is extracted from foods using phosphate buffer, captured on heparin affinity columns to remove other components, and then eluted with high-salt buffer. The isolated bLF is subsequently quantified by reversed-phase HPLC against an external standard bLF. Zhang et al. demonstrated the application of this method to several food matrixes, including sterilized milk, modified fermented milk, vitamin-fortified drinks, infant formula, infant rice cereal, adult formula, and protein powder. The recovery of spiked bLF in these food matrixes varied with matrix type, ranging from 80 to 96%.

Although Zhang’s HPLC-based LF analysis method using heparin affinity columns is practical and reproducible, its performance remains matrix-dependent, which poses a challenge for broader applicability. Here, we report a modified heparin affinity column-based bLF analysis method employing an improved extraction buffer system with optimized pH and a combination of chemical additives. The performance of the optimized extraction buffer was evaluated through spiking and recovery tests in various dairy product matrixes, and bLF recovery was demonstrated to be more effective and versatile compared to the standard extraction buffer (phosphate buffer).

## Experimental

### Apparatus

- *HPLC.*—HPLC was performed on an LC-2050C 3D system (Shimadzu, Kyoto, Japan) with a UV detector.
- *Reversed-phase HPLC column.*—A Waters XBridge Protein BEH C4 column (300 Å, 3.5 µm, 4.6 × 150 mm) was used (Waters, Milford, MA, USA).
- *Milli-Q water purification system.*—A Milli-Q^®^ IQ 7000 was used (Merck Millipore, Darmstadt, Germany).
- *Shaker.*—A universal shaker SHK-U4 was used (IWAKI, Tokyo, Japan).
- *Centrifuge.*—High speed refrigerated micro centrifuge MDX-310 was used (Tomy Digital Biology, Tokyo, Japan).
- *Water bath.*—An HOA-50A oil bath was used (ASONE, Osaka, Japan).
- *Electronic balance.*—A BXPR204 balance was used (METTLER TOLEDO, Greifensee, Switzerland).
- *Heparin chromatography system.*—An ÄKTA start chromatography system was used (Cytiva, Uppsala, Sweden).
- *Vortex mixer.*—A Vortex-Genie 2 was used (Electro Scientific Industries, Portland, OR, USA).

### Reagents

- *Water.*—Purified in house using the Milli-Q system (Merck Millipore, Darmstadt, Germany).
- *Bovine lactoferrin (bLF).*—Food Additives Test grade (FUJIFILM Wako Pure Chemical, Osaka, Japan).
- *Disodium hydrogen phosphate (Na_2_HPO_4_).*—Analytical Grade (FUJIFILM Wako Pure Chemical).
- *Phosphoric acid.*—Analytical grade (FUJIFILM Wako Pure Chemical).
- *Urea.*—Analytical grade (FUJIFILM Wako Pure Chemical).
- *Ethanol.*—Analytical grade (FUJIFILM Wako Pure Chemical).
- *Acetonitrile.*—HPLC grade (FUJIFILM Wako Pure Chemical).
- *Trifluoroacetic acid.*—Analytical grade (FUJIFILM Wako Pure Chemical).
- *Sodium chloride (NaCl).*—Analytical grade (Kanto Chemical, Tokyo, Japan).
- *Sodium hydroxide.*—Analytical grade (Kanto Chemical).
- *EDTA (0.5 M, pH 8.0).*—Analytical grade (Merck Millipore, Burlington, MA, USA).
- *Tween 20.*—Analytical grade (Tokyo Chemical Industry, Tokyo, Japan).

### Buffer Preparation

The buffer compositions used for bLF extraction and isolation were as follows: standard extraction buffer [as reported in Zhang’s study (14)]: 200 mM Na_2_HPO_4_ (pH 8.0, adjusted with phosphoric acid); optimized extraction buffer: 200 mM Na_2_HPO_4_ with 2.0 M urea, 80 mM NaCl, 4.0 mM EDTA, and 0.16% (w/v) Tween 20 (pH 6.0, adjusted with phosphoric acid); binding buffer (for heparin column conditioning): 200 mM Na_2_HPO_4_ (pH 8.0, adjusted with phosphoric acid); and elution buffer: 50 mM Na_2_HPO_4_ with 1.0 M NaCl (pH 8.0, adjusted with phosphoric acid).

### Spiking and Extraction of bLF

The amount of bLF used in all experiments was adjusted according to its purity (97.1%), as determined by reversed-phase HPLC analysis provided by the manufacturer.

**Figure 1.**
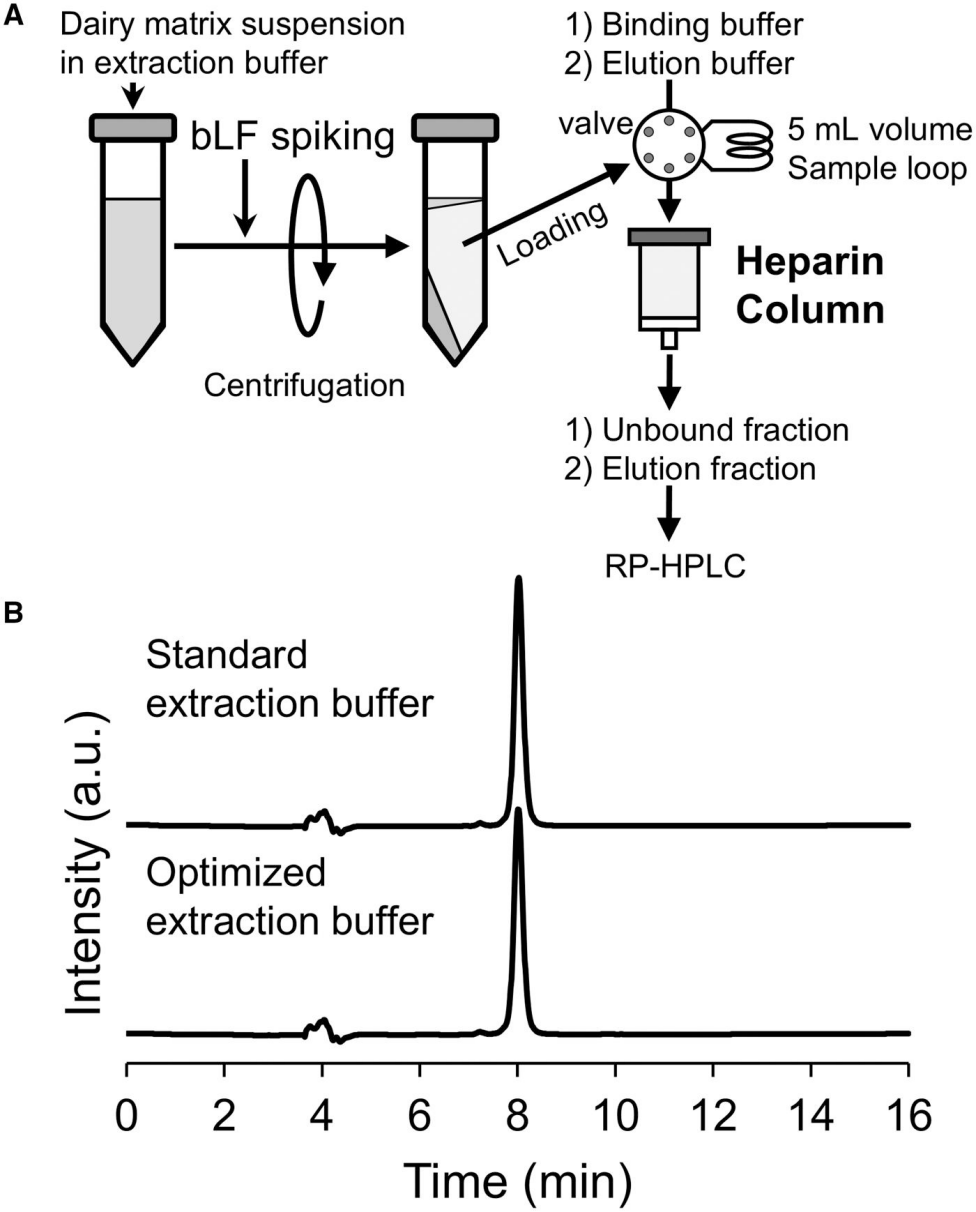
(A) Experimental scheme: bLF was added to the food matrix suspension in the extraction buffer and centrifuged. The aqueous supernatant, between the insoluble precipitate and the upper fat layer, was loaded into a sample loop and applied to a heparin column. The eluted bLF was analyzed by reversed-phase HPLC (RP-HPLC). See *Experimental* section for details. (B) RP-HPLC Chromatogram: chromatogram of bLF isolated from infant formula with partially hydrolyzed proteins by heparin affinity method, using a standard extraction buffer (upper) and an optimized extraction buffer (lower). The peak at 8 min corresponds to bLF, and its area was used for quantification.

The bLF spiking and recovery test was conducted as follows (Figure 1A). Accurately weighed bLF powder (approximately 100, 250, 500, and 1000 mg) was transferred into a 100 mL volumetric flask and diluted to a final volume of 100 mL with water to prepare 1, 2.5, 5, and 10 mg/mL bLF solutions, respectively. The actual weight of bLF powder was used to correct the concentration of each solution, which was then applied in recovery yield calculation. One gram of the dairy products matrix was measured in a 15 mL tube and suspended or dissolved in 11.5 mL pre-warmed (40°C) extraction buffer (standard or optimized). To this solution, 500, 400, and 500 μL bLF solutions at concentrations of 1, 2.5, and 10 mg/mL, respectively, were added, resulting in spiking levels of 50, 100, and 500 mg bLF per 100 g matrix, respectively. For a spiking level of 25 mg bLF per 100 g, 1.5 g of the dairy products matrix was used with 375 μL 1 mg/mL bLF solution. The spiked solution was mixed with a vortex mixer for 30 s, shaken for 60 min at 150 rpm, and centrifuged at 8000*g* for 20 min at 4°C. The aqueous supernatant, avoiding the upper fat layer, was collected into a 25 mL volumetric flask and diluted to 25 mL with extraction buffer. For the double extraction procedure, the first extraction was conducted as described above, followed by the addition of 11 mL extraction buffer to the centrifuge tube. The insoluble residue was suspended using a vortex mixer for 10 s, shaken for 20 min, centrifuged, and the aqueous supernatant was combined with the first in the volumetric flask. For the triple-extraction procedure, 8 mL extraction buffer was used in the first and second steps, and 7 mL in the third step.

To prepare heat-denatured bLF, 1 mL 5 mg/mL bLF solution was transferred into a 1.5 mL microtube and heated in a water bath at 90°C for 1 h. After heating, the sample was cooled to room temperature and diluted 5-fold with Milli-Q water using a volumetric flask to obtain a 1 mg/mL solution. This heat-treated bLF solution was then used for spiking experiments in the same manner as the non-heated bLF described above, ensuring a spiking level of 50 mg per 100 g matrix.

### Isolation of bLF Using a Heparin Column

Isolation of bLF from the extracted solution was performed using an ÄKTA start chromatography system equipped with a heparin affinity column (1 mL HiTrap Heparin HP column, Cytiva, Uppsala, Sweden) at a flow rate of 1.0 mL/min. The column was equilibrated with 5 mL binding buffer. Ten milliliters of the extracted bLF solution was injected into a 5 mL sample loop to ensure complete filling. The bLF solution was then introduced into the column, followed by washing with 10 mL binding buffer. Elution was performed by applying 5 mL elution buffer to the column, and the latter 4.5 mL of the eluate was collected into a 5 mL volumetric flask, diluted to volume with elution buffer, and used as the isolated bLF solution.

### HPLC Analysis of Isolated bLF

If spiked bLF was recovered with 100% efficiency during extraction and isolation, the resulting concentrations would be 15, 20, 40, and 200 μg/mL for spiking levels of 25, 50, 100, and 500 mg bLF per 100 g matrix, respectively. These theoretical values were used as denominators in recovery yield calculations for subsequent HPLC analysis. The isolated bLF was analyzed using reversed-phase HPLC on an LC-2050C 3D HPLC system equipped with a Waters XBridge Protein BEH C4 column (300 Å, 3.5 µm particle size, 4.6 × 150 mm, Waters) at 35°C. The elution of bLF was monitored using a UV spectrophotometer (280 nm). The isolated bLF solution eluted from the heparin affinity column was filtered through a 0.45-μm cellulose membrane (Pall Co., New York, USA), and 50 μL was injected onto the column. The elution was performed at a flow rate of 0.5 mL/min using two mobile phases: (A) water containing 0.1% (v/v) trifluoroacetic acid and (B) acetonitrile containing 0.1% (v/v) trifluoroacetic acid. The gradient program was as follows: 0–5 min, linear gradient from 70% A and 30% B to 55% A and 45% B; 5–10 min, linear gradient to 40% A and 60% B; 10–12 min, linear gradient to 30% A and 70% B; 12–14 min, linear gradient to 70% A and 30% B; 14–16 min, isocratic 30% A and 70% B. The bLF peak was detected (at about 8 min, Figure 1B) and integrated with Lab Solutions software (Shimadzu). Quantification was based on a linear calibration curve constructure from standard bLF solutions (10, 20, 50, 100, 150, and 250 μg/mL) with a coefficient of determination (*r*^2^) of at least 0.995. A 1/*x* weighting factor, where *x* represents the bLF concentration of the calibration standards, was applied to minimize bias and improve accuracy at low concentrations, although this approach may increase error at high concentrations.

All measurements were performed in triplicate, and statistical analysis was performed using Microsoft Excel.

## Results

### Verification of Heparin Method with Reported Conditions

One of the major applications of bLF is its use as a supplement in infant formula. The applicability of the reported heparin method (14) for bLF quantification in infant formula products was verified through a bLF spiking and recovery test (Figure 1A and Table 1, Nos 1–5). The results showed high recovery rates of 80.4–86.7% for standard infant formula at a spiking level of 50–500 mg/100 g, but only 42.0 and 53.7% for distinct formulas containing partially hydrolyzed proteins under the conditions described in the reported heparin method (14). Low recovery rates were also found in several other matrixes (Table 1). This matrix-dependent reduction of bLF recovery could possibly be attributed to complex interactions between food components and bLF. Some components may associate with the surface of bLF molecules, reducing their accessibility to heparin resin and lowering binding efficiency. Furthermore, when bLF is trapped by insoluble food components through such interactions, its extraction into the aqueous solution is diminished. Therefore, reducing or eliminating these interactions could improve bLF recovery using the heparin method. To address this, the optimal composition of the extraction buffer for suspending and dissolving bLF-supplemented dairy products was evaluated.

**Table 1.** Comparison of the standard and optimized extraction buffer

No.^a^	Extraction buffer	Spiking level, mg/100 g	Standard (1–11)	Optimized (12–22)
	matrix		Recovery, %	
1, 12	Infant formula	500	86.7 ± 0.1	101.1 ± 1.0
2, 13		100	85.2 ± 2.6	99.4 ± 3.3
3, 14		50	80.4 ± 2.7	96.7 ± 2.9
4, 15	Infant formula with partially hydrolyzed proteins (Product 1)	500	42.0 ± 1.3	92.3 ± 2.2
5, 16	Infant formula with partially hydrolyzed proteins (Product 2)	500	53.7 ± 1.4	104.2 ± 2.5
6, 17	Milk	500	92.7 ± 0.8	102.6 ± 0.9
7, 18	Yogurt	500	96.1 ± 2.7	102.9 ± 0.4
8, 19	Adult nutrition powder	500	82.5 ± 5.3	102.7 ± 2.7
9, 20		100	82.0 ± 4.2	92.6 ± 7.1
10, 21		50	95.4 ± 5.0	97.9 ± 0.4
11, 22	Adult nutrition powder with cocoa	500	64.2 ± 6.7	98.0 ± 2.0

a Numbers 1–11 correspond to results obtained using the standard extraction buffer for each matrix. Numbers 12–22 correspond to results obtained using the optimized extraction buffer for the same matrixes.

### Development of Improved bLF Extraction Buffer with Optimized Composition

An improved extraction buffer was developed using infant formula with partially hydrolyzed proteins as the test matrix, as this formulation exhibited low recovery rates with the standard extraction buffer (Table 1). To address this issue, the buffer composition was systematically modified based on the physicochemical properties of bLF and its interaction with heparin (Table 2).

**Table 2.** Evaluation of extraction buffer pH and additives to improve the recovery rate of spiked bLF (500 mg/100 g) using an infant formula with partially hydrolyzed proteins (Product 1) matrix

No.^a^	Na_2_HPO_4_, mM	pH	Urea, M	NaCl, mM	EDTA, mM	Tween 20, % (w/v)	Recovery, %
1	200	6	—^b^	—	—	—	56.2 ± 4.1
2	200	6	1	—	—	—	63.6 ± 0.6
3	200	6	1.5	—	—	—	68.0 ± 2.8
4	200	6	2	—	—	—	69.3 ± 3.8
5	200	6	2	80	—	—	83.7 ± 4.0
6	200	6	2	—	4	—	77.1 ± 10.4
7	200	6	2	—	—	0.16	87.2 ± 4.1
8	200	6	2	80	4	—	81.6 ± 0.5
9	200	6	2	80	—	0.16	87.8 ± 2.7
10	200	6	2	80	4	0.16	93.8 ± 3.6
11	200	7	2	80	4	0.16	87.3 ± 3.7
12	200	8	2	80	4	0.16	83.9 ± 2.0
13	200	6	—	80	4	0.16	84.6 ± 1.7

a Numbers indicate different extraction buffer compositions tested for infant formula with partially hydrolyzed proteins (Product 1), as detailed in the table.

b — = Additive was not added.

The first parameter examined was pH. The interaction between heparin, which contains strongly acidic sulfate groups, and proteins is influenced by the net charge of the protein, which varies with pH. Under mildly acidic conditions, proteins carry more positive charges, thereby strengthening electrostatic interactions with heparin. Previous studies demonstrated that bLF binds more strongly to heparin at around pH 6.0 than at neutral pH (12, 17, 18). Based on this evidence, the extraction buffer was adjusted to pH 6.0, which increased recovery to 56.2% (Table 2, No. 1) compared with the original pH 8.0 buffer (42.0%, Table 1, No. 4), although the improvement was still insufficient.

Next, the effect of urea was investigated. Urea acts as a chaotropic agent that enhances protein solubility and prevents aggregation by disrupting hydrophobic interactions between biomolecules. However, excessively high concentrations of urea can denature proteins. Because analytical methods for bLF in food products are designed to quantify the native form, avoiding denaturation is essential. Human lactoferrin (hLF) is reported to denature at urea concentrations above 3 M (19), and bLF at concentrations above 2 M of guanidine hydrochloride (20), which is a stronger denaturant than urea (21). Therefore, urea concentrations below 2 M were tested to enhance bLF recovery while maintaining its native structure; increasing the urea concentration up to 2.0 M further improved recovery (Table 2, Nos 2–4).

Because bLF may associate with food components through non-specific electrostatic interactions, salts were added to suppress these interactions. However, excessively high salt concentrations, such as those in 1 M NaCl elution buffers for heparin affinity chromatography, can disrupt bLF–heparin binding. Previous studies have reported that NaCl at concentrations of 80–120 mM promotes the binding of hLF to heparin, whereas multivalent metal ions such as Ca^2+^, Cu^2+^, Zn^2+^, Fe^2+^, and Fe³^+^ inhibit the binding of bLF (12). Accordingly, NaCl at 80 mM and the chelating agent EDTA at 4 mM to sequester multivalent metal ions were evaluated in combination with 2 M urea as the base condition. Both additives provided additional improvements in recovery (Table 2, Nos 5 and 6).

Interactions between bLF and lipids in the matrix were also considered. Non-ionic surfactants such as Tween 20 are effective in dissociating hydrophobic substances from protein surfaces. Therefore, Tween 20 at 0.16%, a commonly used concentration, was added to the buffer containing 2 M urea, and this further improved recovery (Table 2, No. 7).

Based on these findings, their combined effect was evaluated at pH 6.0 in the presence of 2 M urea (Table 2, Nos 8–10). Although the effects of individual additives varied, their combined use overall enhanced recovery. The inclusion of Tween 20 with NaCl provided a greater improvement than NaCl alone, whereas the addition of EDTA to NaCl did not yield a synergistic effect. However, the simultaneous incorporation of all three additives—NaCl, EDTA, and Tween 20—produced a marked increase in recovery, and the buffer containing all four components (2 M urea, 80 mM NaCl, 4 mM EDTA, and 0.16% Tween 20) achieved the highest recovery rate (93.8%, Table 2, No. 10). To verify the influence of pH under the optimized additive conditions, the buffer containing all four components was further tested at pH 7.0, and 8.0 (Table 2, Nos 11 and 12). Recovery decreased progressively with increasing pH, confirming that pH 6.0 is the most suitable condition. This condition, containing all four additives, was adopted as the optimized extraction buffer for the experiments described in this study. Also, omission of urea from this formulation substantially reduced its effectiveness, indicating that urea is essential for high recovery (Table 2, No. 13).

Figure 1B shows the reversed-phase HPLC chromatogram of bLF spiked into infant formula with partially hydrolyzed proteins, extracted using both the standard and optimized extraction buffers, and isolated with a heparin affinity column. Both chromatograms exhibited a single bLF peak with identical retention times.

### Applicability of the Optimized Extraction Buffer

The applicability of the optimized extraction buffer for recovering bLF from various dairy product matrixes was evaluated. A spiking and recovery test was performed using both the standard and optimized extraction buffers. The optimized extraction buffer demonstrated consistently higher recovery rates across all matrixes than those of the standard buffer (Table 1). These results confirmed the improved applicability and versatility of the optimized extraction buffer.

### Effect of Extended Extraction Procedures on Dairy Products with Lower bLF Content

**Figure 2.**
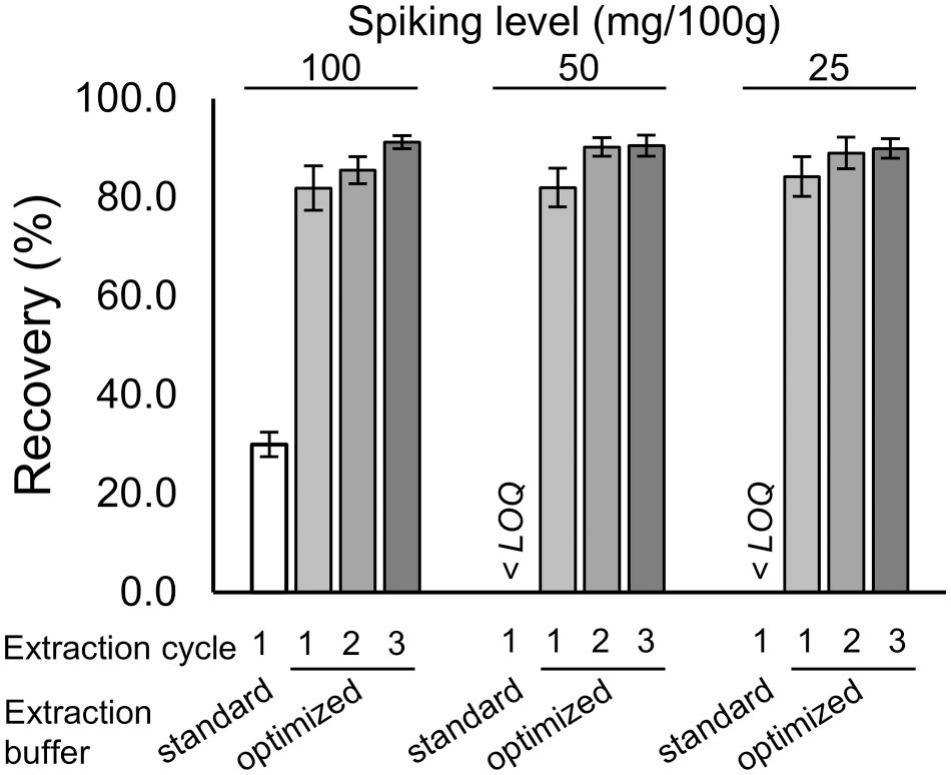
Recovery of bLF at low spiking level (<100 mg/100 g) in infant formula with partially hydrolyzed proteins (Product 1) using standard phosphate buffer and optimized buffer. For optimized buffer, single, double, and triple extraction cycles were evaluated.

When the spiking level was reduced below 100 mg/100 g, the recovery of bLF from infant formula with partially hydrolyzed proteins decreased markedly when using the standard extraction buffer with the heparin method. Even at such low levels, the optimized extraction buffer improved recovery, although the rates did not reach those obtained at higher spiking levels, (e.g., 500 mg/100 g). As described in a previous study (16), repeated extraction of the post-centrifugation residue was carried out using the infant formula with partially hydrolyzed proteins (Product 1). Double or triple extractions with the optimized extraction buffer significantly improved recovery (Figure 2). The reduction in recovery observed with the standard buffer at low bLF levels was also observed in other matrixes. In these cases, multiple extractions with the optimized extraction buffer effectively enhanced recovery rates (Table 3).

**Table 3.** Comparison of the standard buffer used in a single extraction and the optimized buffer used in triple extractions.

No.^a^	Matrix	Spiking level, mg/100 g	Recovery, %	
			Standard buffer (1–4)	Optimized buffer (5–8)
1, 5	Infant formula with partially hydrolyzed proteins (Product 2)	100	41.6 ± 1.3	97.8 ± 0.2
2, 6		50	<LOQ	95.6 ± 1.5
3, 7	Adult nutrition powder with cocoa	100	74.2 ± 1.3	95.2 ± 3.7
4, 8		50	71.8 ± 0.6	94.1 ± 0.6

a Numbers 1–4 correspond to results obtained using the standard buffer in single extraction for each matrix. Numbers 5–8 correspond to results obtained using the optimized buffer in triple extractions for the same matrixes.

### Exclusion of Heat-Denatured bLF from Quantification

**Figure 3.**
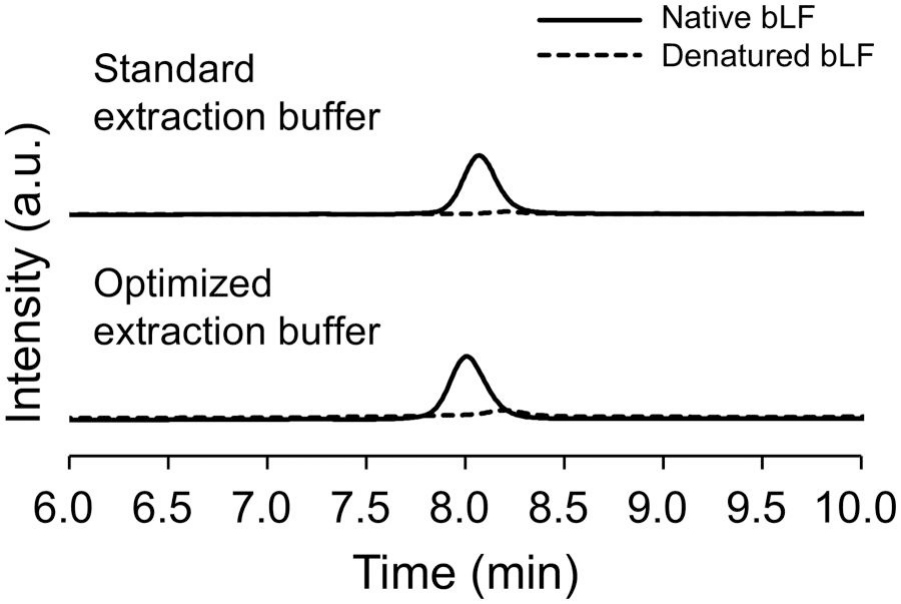
RP-HPLC Chromatogram: chromatogram of native (solid line) and heat-denatured (dashed line) bLF isolated from infant formula by heparin affinity method, using the standard extraction buffer (upper) and the optimized extraction buffer (lower).

The biological functions of bLF depend on its three-dimensional structure, which is lost upon heat denaturation or other destabilizing factors. Therefore, it is desirable to exclude denatured bLF from quantification in food matrixes. The reported heparin method with a standard extraction buffer demonstrated the exclusion of heat-denatured bLF. This property was also evaluated for the optimized extraction buffer. Figure  3 presents reversed-phase HPLC chromatograms of solutions eluted from the heparin column, showing that denatured bLF was not detected with either the standard or optimized extraction buffer.

## Discussion

When bLF was spiked into specific matrixes, its quantification using the heparin method with standard extraction buffer was compromised by low recovery. We hypothesized that interactions between bLF and certain matrix components led to molecular associations that complicated accurate quantification. Therefore, the study explored buffer compositions to weaken these interactions, maintain bLF in a non-associated state with matrix components, and improve its binding to the heparin column. Urea, surfactants, salts, and chelating agents each contributed to higher recovery rates in the spiking tests. These effects were additive, suggesting that the interactions between bLF and food components are complex and heterogeneous. Furthermore, the reversed-phase HPLC chromatograms (Figure 1B) showed that bLF, isolated on a heparin affinity column using the optimized extraction buffer exhibited a retention time comparable to that obtained with the standard buffer, confirming effective isolation from major interfering components of the dairy matrix and supporting accurate quantification. Figure  3 confirmed that denatured bLF was not detected with either the standard or optimized extraction buffer, indicating that the heparin method using the optimized buffer selectively detects native bLF. Adjusting additive formulations in this way expanded the applicability of the developed buffer while preserving the exclusion of heat-denatured bLF. Moreover, the association of bLF with food components may also hinder other analytical methods based on antibody recognition, such as ELISA and biosensors. In such cases, the buffer optimization strategy developed here could similarly enhance analytical performance. Validation using incurred samples will be required to establish the robustness and applicability of the optimized buffer under practical conditions.

## Conclusions

In this study, we optimized an extraction buffer for bLF quantification using the heparin method by refining its composition and operating conditions. Improvements were achieved through a systematic selection of additives informed by published literature and biomolecular interaction insights. Furthermore, we assessed the impact of multiple extraction steps, demonstrating that additional extractions enhance recovery when initial yields are low.

Based on these findings, we recommend the following optimized conditions for bLF quantification: extraction buffer containing 200 mM Na_2_PO_4_ (pH 6, adjusted with phosphoric acid), 2 M urea, 80 mM NaCl, 4 mM EDTA, and 0.16% (w/v) Tween 20. For extraction steps, one extraction is sufficient for high bLF concentrations (>100 mg/100 g), whereas two to three extractions should be considered for lower concentrations.

Overall, this work establishes a more accurate, versatile, and broadly applicable approach for bLF quantification using the heparin method.

## CRediT Author Statement

Tomoaki Fukushima (Data curation [Lead], Investigation [Equal], Validation [Lead], Writing—original draft [Equal], Writing—review & editing [Supporting]), Keisuke Yamamoto (Conceptualization [Equal], Data curation [Equal], Investigation [Equal], Methodology [Equal], Validation [Equal]), Yukino Sato (Data curation [Equal], Investigation [Equal], Validation [Equal], Writing—review & editing [Supporting]), Tatsuya Nojima (Conceptualization [Lead], Methodology [Lead], Visualization [Lead], Writing—original draft [Equal], Writing—review & editing [Lead]), Mirei Odaka (Conceptualization [Lead], Data curation [Equal], Investigation [Equal], Validation [Equal], Writing—review & editing [Equal]), Norichika Nishida (Supervision [Equal], Writing—review & editing [Supporting]), and Hiroshi Ochi (Supervision [Equal])
